# Enhanced magneto-optical rotation of probe field in thermal medium via spontaneous generated coherence

**DOI:** 10.1038/s41598-022-13374-z

**Published:** 2022-06-13

**Authors:** Saddaf Sultan, Hazrat Ali, Rafi Ud Din, M. Imtiaz Khan, Bin Amin, Muhammad Shafiq, Mahidur R. Sarker, Sawal Hamid Md Ali

**Affiliations:** 1 Department of Physics, Abbottabad University of Science and Technology, Havelian, Pakistan; 2 Department of Applied Physical and Material Sciences, University of Swat, Swat, Pakistan; 3 Institute of IR 4.0, Unverisity Kebangsaan Malaysia, 43600 Bangi, Malaysia; 4 Department of Electrical, Electronic and Systems Engineering, Faculty of Engineering and Built Environment, University Kebangsaan Malaysia, 43600 Bangi, Malaysia

**Keywords:** Optics and photonics, Physics

## Abstract

A four-level double lambda closed atomic configuration is considered to study the polarization plane rotation of the probe beam through cold as well as thermal Rb$^{87}$ atomic medium by varying the spontaneously generated coherence (SGC). Magnetic field and strong coupling field are applied to the atomic configuration. The light-matter interaction leads to enhanced the magneto-optical rotation. The intensity of the applied fields plays promising role in the generation and enhancement of birefringence. It ultimately enhances the polarization plane rotation of the probe beam in the Doppler medium. In the presence of both SGC and Doppler broadening effects, the optical rotation and transmission of the weak light beam are modified and controlled as well, which have potential applications in magnetometery and laser frequency stabilization.

## Introduction

Interaction between matter and plane-polarized light has wide range of applications in modern technology like sensitive magnetometers^1,2^, occurrence of parity^3^, magneto-optical switching^4^, time-reversal violation^5^, laser frequency stabilization^6,7^, optical isolators^8,9^, current sensors^10,11^, optical limitation^12,13^, spectroscopy^14–16^, magneto-optical rotation and optical filters^17–20^. One such interaction leads to Magneto-optical rotation (MOR), a phenomenon where the plane of polarized light is rotated while passing through an anisotropic medium subjected to a magnetic field^21^. This phenomenon is attracting tremendous interest due to its wide technological applications including atomic clocks^22^, magnetometry^2,23^, atomic filters^20^, and optical limitations^18^.

The magnetic field induces asymmetry in a medium by creating Zeeman sublevels^24^ and can be applied either parallel or perpendicular to the propagation direction of light leading to Faraday rotation and the Voigt effect, respectively^25,26^. Petrosyan et al. not only setup a feasible experimental platform for inducing MOR through a medium but also employed it further for optical magnetometry^27^. The output signal of the MOR depends both on the static magnetic field and light, which allows us to measure non-zero magnetic field accurately^28,29^.The MOR has widely been studied in various media and manipulated with various controlling parameters. To mention a few, the rotation of a probe field was enhanced and controlled by the intensity and relative phase of a driving field through a double V-Type atomic medium^30^. The MOR was significantly varied through a quantum well waveguide with varying length of the well and strength of the magnetic field^31^. In a four-level cold atomic system subjected to a static magnetic field, the MOR was enhanced with the introduction of nonlinearity in the sample^25,32^. In another work, nonlinear MOR of a polarized probe field was investigated in inverted Y-type configuration of atoms interacting with a static magnetic field and Laguerre-Gaussian (LG) beam. It was reported that the LG field induces azimuthal asymmetrical polarization distribution^33^. Moreover, the angular momentum and radius of the LG field was shown to effectively control the asymmetrical nature of the medium and ultimately its optical rotation^34^. Nitrogen-vacancy centre (three-level closed systems) was used to study the effect of an acoustic field on the MOR where a maximum of 90 degrees of rotation was achieved^35^.

In a thermal medium, dielectric function of the material becomes temperature-dependent and the atoms gain extra velocities. One such effect is the Doppler broadening where Fermi observed the broadening of spectral lines due to the distribution of velocities of atoms or molecules^36^. The MOR for a polarized probe field in a four-level Doppler broadened medium was recently evaluated and a significant enhancement of the rotation angle was observed^25^. While investigating temperature-dependent MOR in multiple three-level media, Li et al. observed a 45 degrees rotation at 65 C^37^. Doppler broadening is an efficient technique employed in a wide range of media for different purposes. For example, the effect of inhomogeneous Doppler broadening on slow light in a two-level squeezed vacuum-assisted qubit system was investigated by some of the authors of the present study^38,39^.

Spontaneous emission coherence (SEC) or generated coherence (SGC) is another useful phenomenon which causes additional coherence, arising from degenerate or near-degenerate levels of the system^40^. The coherence is prominent via the spontaneous emission from a single excited to two ground states (lambda type system) or from the two upper excited states to any single ground state (*V* type). Closely spaced levels with no orthogonal dipole matrix elements are the necessary conditions for SEC. The SEC in multilevel atomic media has been used to manipulate their electrical and optical properties^41–49^. In another study, the atom decays spontaneously from a single excited to two grounds eigenstates in the lambda configuration^50^. The spontaneous coherence in V-type atomic configuration arises due to the decay from two excited states to a singlet ground state^51^. Sharp absorption peaks of the propagating field are noticed in a Y-type configuration having SGC that is driven by two control fields^52^.

From the above mentioned literature, we see that no study has yet been reported for the investigation of MOR in a medium with thermal and/or cold atoms under the SGC effect. However, the propagation of an optical beam through four levels of an atomic medium with both Doppler broadening and SGC effects was studied^53^. Therefore, in this article the MOR of polarized probe light is studied in double lambda closed medium by varying both the static magnetic field and generated coherence (SGC) for cold and thermal atoms. We notice an enhanced clock and anti-clockwise MOR with varying the SGC in Doppler broadened assisted medium. The transmission along the *y*- and *x*-axis is controlled and modified through the thermal medium with considering SGC in the system. The results of this study may find applications in polarization spectroscopy, precision measurements and polarization converters.

## Theoretical framework

**Figure 1:**
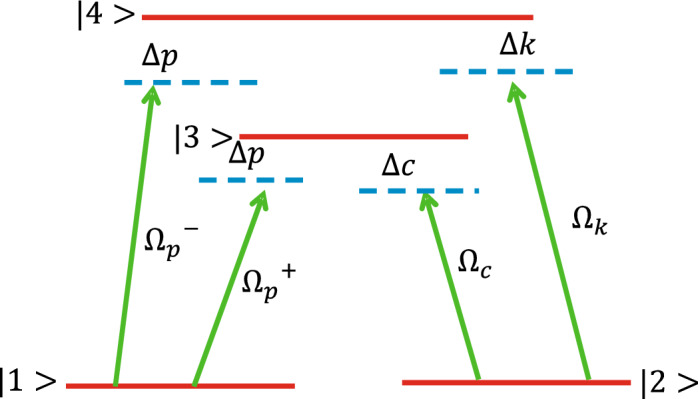
Four level double lambda configurations driven by right/left circularly polarized field having Rabi frequency $(\Omega _{p}^{+}$ / $\Omega _{p}^{-}$ respectively.

A four-level double-lambda type configuration is considered for the MOR of the probe light, as depicted in Fig. 1. The system is an ensemble containing two doubly-degenerate ground states, namely, $\mid {1, m = 1 \rangle }$ and $\mid {2, m = -1 \rangle }$ and two upper excited states $\mid {3, m = 0 \rangle }$ and $\mid {4, m = 0\rangle }$. The system is generated in $D_{2}$ lines of $\hbox {Rb}^{87}$ vapour atomic system, while the transitions occur between $5^2 S_{(1/2)}\iff 5^2 P_{(3/2)}$. The magnetic field ($\vec {B}$ = $B {{\hat{z}}}$) applied in the *z*-direction splits the energy level, i.e., $\Delta B = \frac{m_{s} g_{s} \mu _{B} B}{\hbar }$. Besides, a weak probe field $\vec {E}=xE_{p} e^{i(k_{p}z-\omega _{p}t)} + c.c$, which is linearly polarized, is applied parallel to the magnetic field ($\vec {B}$ = $B {{\hat{z}}}$) in the closed medium. Right and left circularly polarized fields collectively constitute the applied linearly polarized probe field. The right (left) circular component with Rabi frequency $\Omega _{p}^{+}$ ($\Omega _{p}^{-}$) causes transition $\mid {3}\rangle \iff \mid {1}\rangle$ ${(}\mid {4}\rangle \iff \mid {1}\rangle {)}$ where $\Omega _{p}^{+}=E_{+} (\frac{\left( \mathbf {\mu }_{31}. {\hat{\varepsilon }}_{+}\right) }{\hbar })$ and $\Omega _{p}^{-}=E_{-} (\frac{\left( \mathbf {\mu }_{41} \cdot {\hat{\varepsilon }}_{-}\right) }{\hbar })$. Another coupling field with frequency $\Omega _{k}$ is applied between staes $|2\rangle$ and $| 4\rangle$ having Rabi oscillation $\Omega _{k}=\frac{\left( \mathbf {\mu }_{24}, {\hat{\varepsilon }}_{k}\right) }{\hbar } E_{k}$. $E_{+}= E_{-}=\frac{E_{p}}{\sqrt{2}}$, $\left| \mu _{31}\right| =\left| \mu _{41}\right|$ and $\varepsilon _{i}(i=\pm , c)$ being directional vectors of right/left circularly polarized lights and coupling field. The splitting of Zeeman energy levels occurs in $|3\rangle$ and $| 4\rangle$ and can be obtained by $\hbar \Delta _{B}=m_{s} g_{s} \mu _{B} B$, $\mu _{B}$ ( $g_{s}$) being the Bohr’s magneton (Lande’s factor) and $m_{s}=\pm 1$ is quantum number (magnetic) of the respective sub-levels of excited states. Applying the electric dipole approximation as well as rotating frame approximation, the interaction picture of Hamiltonian is given by:

1 $$\begin{aligned} H= & {} -\hbar \left[ \Omega _{P}^{-} e^{-i(\Delta _{p_+}-\Delta B) t}|4\rangle \langle 1|+\Omega _{p}^{-} e^{-i(\Delta _{p_+}-\Delta B) t}\right. |1\rangle \langle 4| +\Omega _{p}^{+} e^{-i(\Delta _{p_-}-\Delta B) t}|3\rangle \langle 1|\nonumber \\&+ \Omega _{p}^{+} e^{-i(\Delta _{p_-}-\Delta B) t}|1\rangle \langle 3| +\Omega _{c} e^{-i(\Delta c-\Delta B) t} |3\rangle \langle 2|+\Omega _{c} e^{-i(\Delta c-\Delta B) t} \quad |2\rangle \langle 3| \nonumber \\&\quad +\Omega _{k} e^{-i(\Delta k-\Delta B) t} |4\rangle \langle 2|+\Omega _{k} e^{-i(\Delta k-\Delta B) t} \quad |2\rangle \langle 4|+ c.c. \end{aligned}$$

Here, $\Delta _{p_+}=\omega _{41}-\omega _{p_+}$, $\Delta _{p_-}=\omega _{31}-\omega _{p_-}$ and $\Delta _{c}=\omega _{32}-\omega _{c}$ are the detunings of right, left polarized components and control field with associated central electronic transition frequencies. The density matrix equations on employing liovolli’s equation of the double-lambda (four-level) closed configuration are obtained as

2 $$\begin{aligned} {{\dot{\rho }}}_{31}= & {} (i\Delta _p+i\Delta _B - \gamma _{31})\rho _{31}+i\Omega _{\rho ^{+}}(\rho _{11}-\rho _{33}) + i\Omega _c \rho _{21}\nonumber \\&-i\Omega _{\rho ^{-}} \rho _{34}, \nonumber \\ {{\dot{\rho }}}_{21}= & {} [(i\Delta _p +i\Delta _B- i\Delta _c)-\gamma _{21}]\rho _{21}+i\Omega _c \rho _{31}+i\Omega _k \rho _{41}-\nonumber \\&i\Omega _{\rho ^{+}}\rho _{23}-i\Omega _{\rho ^{-}}\rho _{24},\nonumber \\ {{\dot{\rho }}}_{41}= & {} (-i\Delta _\rho - \gamma _{41})\rho _{41}+(i\Omega _{\rho ^{-}}(\rho _{11}-\rho _{44}))+ \nonumber \\&i\Omega _k \rho _{21}-i\Omega _{\rho ^{+}}\rho _{43}-i\Omega _{\rho ^{-}}\rho _{44},\nonumber \\ {{\dot{\rho }}}_{11}= & {} -\gamma _{11}\rho _{11}+i\Omega _{\rho ^{+}}\rho _{31}+i\Omega _{\rho ^{+}}\rho _{41}-\nonumber \\&i\Omega _{\rho ^{+}}\rho _{13}-i\Omega _{\rho ^{-}}\rho _{14}, \nonumber \\ {{\dot{\rho }}}_{12}= & {} (-i\Delta _c + i\Delta _p -i\Delta _B-\gamma _{12})\rho _{12}+i\Omega _{\rho ^{+}}\rho _{32}+i\Omega _{\rho ^{-}}\nonumber \\&\rho _{42}-i\Omega _c \rho _{13}-i\Omega _k \rho _{14},\nonumber \\ {{\dot{\rho }}}_{13}= & {} (-i\Delta _p -i\Delta _B-\gamma _{13})\rho _{13}-i\Omega _{\rho ^{+}}\rho _{33}+i\Omega _{\rho ^{-}}\rho _{43}+\nonumber \\&i\Omega _{\rho ^{+}}\rho _{11}-i\Omega _c \rho _{12},\nonumber \\ {{\dot{\rho }}}_{14}= & {} (-i\Delta _p +i\Delta _B-\gamma _{14})\rho _{14}+i\Omega _{\rho ^{+}}\rho _{34}+i\Omega _{\rho ^{-}}\rho _{44}-\nonumber \\&i\Omega _{\rho ^{-}}\rho _{11}-i\Omega _k \rho _{12}, \nonumber \\ {{\dot{\rho }}}_{22}= & {} -\gamma _{22}\rho _{22}+i\Omega _c \rho _{32}+i\Omega _k \rho _{42}-i\Omega _c \rho _{23}-\nonumber \\&i\Omega _k \rho _{24}, \nonumber \\ {{\dot{\rho }}}_{23}= & {} (-i\Delta _c-\gamma _{23})\rho _{23}+i\Omega _c \rho _{33}+i\Omega _k \rho _{43}-\nonumber \\&i\Omega _{\rho ^{+}}\rho _{21}-i\Omega _c \rho _{22}, \nonumber \\ {{\dot{\rho }}}_{24}= & {} (i\Delta _c-\gamma _{24})\rho _{24}+i\Omega _c \rho _{34}+i\Omega _k (\rho _{44}-\rho _{22})\nonumber \\&-i\Omega _{\rho ^{-}}\rho _{21}, \nonumber \\ {{\dot{\rho }}}_{32}= & {} (-i\Delta _c-\gamma _{32})\rho _{32}+i\Omega _{\rho ^{+}}\rho _{12}+i\Omega _c\rho _{22}-\nonumber \\&i\Omega _c \rho _{33}-i\Omega _k \rho _{34}, \nonumber \\ {{\dot{\rho }}}_{33}= & {} -\gamma _{33}\rho _{33}+i\Omega _{\rho ^{+}}\rho _{13}+i\Omega _c \rho _{23}-i\Omega _{\rho ^{+}} \rho _{31}-\nonumber \\&i\Omega _c\rho _{32}, \nonumber \\ {{\dot{\rho }}}_{34}= & {} -\gamma _{34}{{\dot{\rho }}}_{34}+i\Omega _{{{\dot{\rho }}}^{+}}{{\dot{\rho }}}_{14}+i\Omega _c {{\dot{\rho }}}_{24}-i\Omega _{{{\dot{\rho }}}^{-}} {{\dot{\rho }}}_{31}-\nonumber \\&i\Omega _k {{\dot{\rho }}}_{32}, \nonumber \\ {{\dot{\rho }}}_{42}= & {} (-i\Delta _c-\gamma _{42})\rho _{42}+i\Omega _{\rho ^{-}}\rho _{12}+i\Omega _k \rho _{22}+\nonumber \\&i\Omega _c \rho _{43}-i\Omega _k \rho _{44}, \nonumber \\ {{\dot{\rho }}}_{43}= & {} -\gamma _{43}\rho _{43}+i\Omega _{\rho ^{-}}\rho _{13}+i\Omega _k \rho _{23}-i\Omega _{\rho ^{+}} \rho _{41}-\nonumber \\&i\Omega _c \rho _{42}, \nonumber \\ {{\dot{\rho }}}_{44}= & {} -\gamma _{44}\rho _{44}+i\Omega _{\rho ^{-}}\rho _{14}+i\Omega _k \rho _{24}-i\Omega _{\rho ^{-}} \rho _{41}-\nonumber \\&i\Omega _k \rho _{42}. \end{aligned}$$

In the set of Eq. (2), $\gamma _{31}{(}\gamma _{41}{)}$ is the decay rate of the excited levels $|1\rangle \longrightarrow |3\rangle$ ($|1\rangle \longrightarrow |4\rangle {)}$. The pump or control field in the presence of SGC effect is given by

3 $$\begin{aligned} \Omega c=\Omega _{1} \sqrt{1-p^{2}}, \end{aligned}$$

where *p* denotes the SGC parameter and describes the quantum coherence that arises from different spontaneous emission channels. The value of *p* depends on the alignment of two the dipole moments $\mu _{13}$ and $\mu _{14}$ and may further be defined as

4 $$\begin{aligned} p=\frac{\mu _{13} \cdot \mu _{14}}{\left| \mu _{13} \cdot \mu _{14}\right| } \mid =\cos \theta , \end{aligned}$$

where $\theta$ being angle between the corresponding dipoles. The coherence paprameter *p* arises when the transition occurs between two emission channels $|3\rangle \leftrightarrow | 1\rangle$ and $\mid 4\rangle \leftrightarrow |1\rangle$. There is no quantum interference ($p=0$) for orthogonal dipole moments whereas $p=1$ is the case of maximum quantum interference that occurs for parallel dipole moments. The control field $\left( E_{c}\right)$ can be used to control the angle $\theta$. The control field in the presence of SGC can be defined as

5 $$\begin{aligned} \Omega _{c}=\Omega _{1} \sin \theta =\Omega _{1} \sqrt{1-p^{2}}. \end{aligned}$$

The polarization response of the medium due to the electric field of right circularly polarized light $E_{p^{+}}$ can be calculated as

6 $$\begin{aligned} {P^{+}}=\chi \varepsilon _{o} E_{p^{+}}, \end{aligned}$$

while the left handed response of the medium due to the probe electric field $E_{p^{-}}$ can be written as

7 $$\begin{aligned} {P^{-}}=\chi \varepsilon _{o} E_{p^{-}}. \end{aligned}$$

In the above equations, $P^{+}=2 N \mu _{31} \rho _{31}$ and $P^{-}=2 N \mu _{41}\rho _{41}$. The expressions for left and right handed susceptibilities ($\chi ^{+} and \chi ^{-}$) of the proposed atom-field medium are given as

8 $$\begin{aligned}&\chi ^{+}=\frac{2 N\left| \mu _{31}\right| ^{2} \rho _{31}^{+}}{\Omega _{p} \hbar \varepsilon _{o}}, \end{aligned}$$

9 $$\begin{aligned}&\chi ^{-}=\frac{2 N\left| \mu _{41}\right| ^{2} \rho _{41}^{-}}{\Omega _{p} \hbar \varepsilon _{o}}. \end{aligned}$$

Here, *N* represents atomic number density, $\mu _{31}$ and $\mu _{41}$ are dipole matrix elements, $\rho ^{+}\left( \rho ^{-}\right)$ is the density matrix element for right (left) circularly polarized light.

The atoms in the sample move with thermal velocities that can modify the control and probe filed frequencies. The apparent or Doppler frequencies can replace the effective frequencies of the probe and control fields, i. e., $\omega _{p}^{+}+\zeta k_{p}^{+}v$, $\omega _{p}^{-}+\zeta k_{p}^{-}v, \omega _{c}+\zeta k_{c}v$ and $\omega _{k}^{+}+\zeta k_{k}v$, where $k_{i}$($i=p, c, k$) is the wave vector of probe and driving fields which are assumed to be equal, i.e., $k_{p}=k_{c}=k_{k}$. The parameter $\zeta =\pm 1$ represents the direction of the wave vector, (+) being co-propagation and (-) describe counter propagation of the wave vectors. Similarly, adding $\zeta k_{k}v$ with the detunings of the probe and control fields, i.e, $\Delta _{p}^{+}+\zeta k_{p}^{+}v$, $\Delta _{p}^{-}+\zeta k_{p}^{-}v, \Delta _{c}+\zeta k_{c}v$, and $\Delta _{k}+\zeta k_{k}v$. The coherence term $\rho ^{+} \left( \rho ^{-}\right)$ depends on the thermal velocity of the atoms and hence the modified left (right) handed susceptibility over the Maxwell-Boltzmann distribution as given by

10 $$\begin{aligned}&\chi _{+}^{S G C(b)}=\frac{1}{D \sqrt{2 \pi }} \int _{-\infty }^{+\infty } \chi _{+}^{S G C} e^{\frac{-(k v)^{2}}{D^{2}}} d(k v), \end{aligned}$$

11 $$\begin{aligned}&\chi _{-}^{S G C(b)}=\frac{1}{D \sqrt{2 \pi }} \int _{-\infty }^{+\infty } \chi ^{S G C} e^{\frac{-(k v)^{2}}{D^{2}}} d(k v). \end{aligned}$$

Here, $kD=\sqrt{\frac{2K_{B}T}{mc^{2}}}$ denotes Doppler width, $K_{B}$ is Boltzman constant, T is the temperature, m is the mass of the moving atom, *k* is the wave vector and *v* is the velocity of the thermal medium.

The MOR in the thermal medium in the presence of SGC can be obtained as

12 $$\begin{aligned} \theta =\frac{2\pi }{\lambda }(Re\{\chi _{+}^{SGC(b)}- \chi _{-}^{SGC(b)}\}d, \end{aligned}$$

where *d* is the length of the medium and $\lambda$ the wavelength of the interacting probe beams. The transmission profiles of the probe field along the *x*-axis $T_{x}$ and *y*-axis $T_{y}$ are obtained by the following relations

13 $$\begin{aligned}&T_{y}=\frac{\left| \left( E_{p_{\text{(out } )}}\right) _{y}\right| ^{2}}{\left| E_{p( \text{ in } )}\right| ^{2}}=\frac{1}{4}\left| \exp \left[ \frac{\iota \alpha \ell \chi _{+}}{2}\right] -\exp \left[ \frac{\iota \alpha \ell \chi _{-}}{2}\right] \right| ^{2}, \end{aligned}$$

14 $$\begin{aligned}&T_{x}=\frac{\left| \left( E_{p_{\text{(out } )}}\right) _{x}\right| ^{2}}{\left| E_{p(i n)}\right| ^{2}}=\frac{1}{4}\left| \exp \left[ \frac{\iota \alpha \ell \chi _{+}}{2}\right] +\exp \left[ \frac{\iota \alpha \ell \chi _{-}}{2}\right] \right| ^{2}. \end{aligned}$$

Birefringence or dichroism are induced in the system when a probe light passes through an asymmetric medium. The difference in the dispersion (absorption) of the right and circularly polarized lights makes the medium birefringent (dichroic). The case ${\text {Re}}\left[ S_{+}\right] \ne {\text {Re}}\left[ S_{-}\right]$ and ${\text {Im}}\left[ S_{+}\right] ={\text {Im}}\left[ S_{-}\right]$ is the most preferred when the medium is birefringent. Hence $T_{x}$ and $T_{y}$ can be written as

15 $$\begin{aligned}&T_{y}=\frac{e^{a l \beta }}{4}\left| \frac{\exp \left[ \iota \alpha \ell R e\left[ S_{+}\right] \right] }{2}-\frac{\exp \left[ \iota \alpha \ell R\left[ S_{-}\right] \right] }{2}\right| ^{2}, \end{aligned}$$

16 $$\begin{aligned}&T_{x}=\frac{e^{a l \beta }}{4}\left| \frac{\exp \left[ \iota a l R e\left[ S_{+}\right] \right] }{2}+\frac{\exp \left[ \iota \alpha l R e\left[ S_{-}\right] \right] }{2}\right| ^{2}, \end{aligned}$$

where $\beta$ is positive and $\alpha l \beta \ll 1$.

## Result and discussion

In this part, we study and discuss the MOR and transmission intensities of the left and right handed lights with and without Doppler broadened double-lambda atomic medium under the presence of SGC. We assume the atomic parameters as $\Omega _{+}=\Omega _{-}=\Omega _{p}, \Delta _{p-}=\Delta _{p+}=\Delta , \alpha l= 190\gamma , kD = 50 \gamma$ equivalent to room temperature for rubidium vapors, $N = 5\times 10^{12}$ atoms/cm$^{3}, \Delta _B=20\gamma$ equivalent to 22 G and all other parameters chosen with respect to $\gamma$, where $\gamma = 2\pi \times$6MHz for $D_{2}$ transition of the rubidium atom.

**Figure 2:**
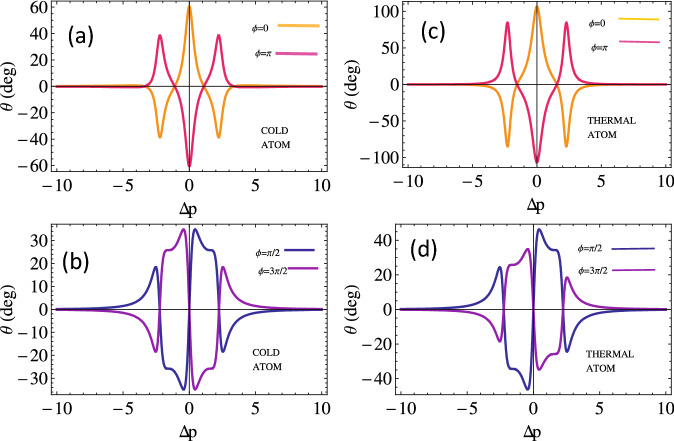
Magneto-optical rotation versus the probe field detuning for cold atoms *KD* = 0 (**a**), (**c**) and thermal atom $kD = 50 \gamma$ (**b**), (**d**) w.r.t relative phase. Other parameters are kept constant to be $\Omega _{1}=2.5, \Omega _{k}=2, p=0.9$, $\Delta _{B}= 10\gamma , \Delta _{c}=0, \Delta _{1}=0$ and $\alpha =190 \gamma$.

**Figure 3:**
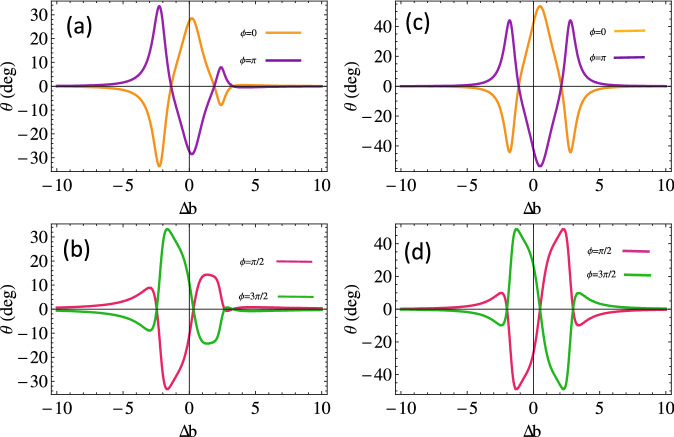
Magneto-optical rotation versus the probe field detuning for cold atom *kD* = 0 (**a**), (**c**) and thermal atom $kD = 50 \gamma$ (**b**), (**d**) w.r.t relative phase. The other parameters are $\Omega _{1}=2.5, \Omega _{k}=2, p=0.9, \Delta _{B}= 10\gamma$, $\Delta _{c}=0, \Delta _{1}=0, \alpha =190 \gamma$.

To investigate magneto-optical (MO) rotation of the light (probe) through the cold and thermal (Doppler broadened) double lambda atomic medium, we plot the rotation angle with probe detuning by considering various values of relative phase $\phi$ as presented in Fig. 2. We notice MO polarization plane rotation of 60 degrees at resonance condition through the cold medium when all the fields are in phase, i.e., $(\phi =0)$. The MO polarization plane rotates counter-clockwise at resonance point by considering the entire fields out of phase in the double lambda atomic medium (see pink curve of Fig. 2a). The MO rotation increases in both directions (clock and anti-clock wise) at resonance point through the Doppler broadened medium and almost notice 1.66 times enhanced MO rotations as depicted in Fig. 2b, leading to an interesting behavior of the MO rotation through the atomic system by considering the relative phases of $\frac{\pi }{2}$ and $\frac{3\pi }{2}$. We notice almost no MO rotation of handed light in the system at $\Delta _{p}=0$ for both phases. The MO rotation of $\pm 35$ degrees at $\Delta _{p}=\pm 0.5 \gamma$ in the cold medium is recorded by taking the relative phase of $90^\circ$ (see blue curve of Fig. 2c). We observe exactly the reverse MO rotation in the medium at phase angle of $\frac{3\pi }{2}$. The enhanced MO rotation of the polarized plane of probe light in the Doppler broadened atomic system is observed for the same set of phase angles (see Fig. 2d). Thus the MO rotation of the light through the medium can be controlled by manipulating the probe detuning.

**Figure 4:**
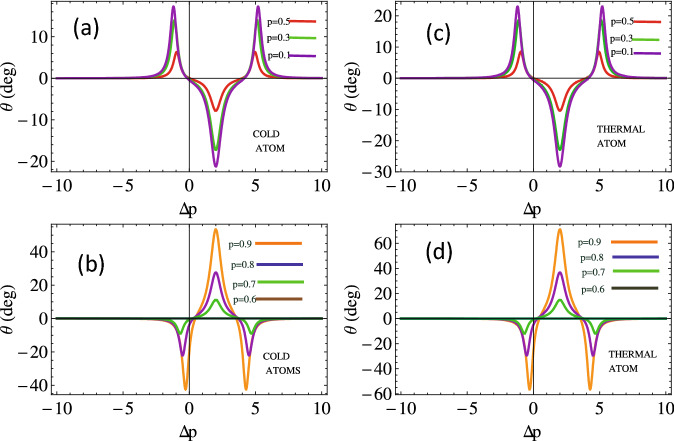
Magneto-optical rotation versus magnetic field detuning for cold atom *kD* = 0 (**a**), (**c**) and thermal atom $kD = 50 \gamma$ (**b**), (**d**) w.r.t relative phase. The other parameters are $\Omega _{1}=2.5, \Omega _{k}=2, p=0.9, \Delta _{p}=0, \Delta _{B}= 10 \gamma , \Delta _{c}=0, \Delta _{1}=0, \alpha =190 \gamma$.

Furthermore, the rotation angle of the polarization plane of the left and right handed light is plotted versus the magnetic field detuning for various values of relative phase angles in cold and thermal atomic medium, as presented in Fig. 3. We observe a $25^\circ$ rotation of the polarization plane (see the orange curve in Fig. 3a) at resonance point for zero relative phases in the cold atom while the polarization plane rotates $180^\circ$ and its peak shifts to $-25^\circ$ when all the fields are out of phase $(\phi =\pi )$. The MOR gets smaller for large positive and negative magnetic field detuning. The MOR is observed almost doubled through Doppler broadened medium in both clockwise and anticlockwise directions (see Fig. 3c). MOR in both cold and thermal medium is studied for the relative phase of $\frac{\pi }{2}$ and $\frac{3\pi }{2}$ (depicted in Fig. 3b, d). Here, we notice that MO rotation of probe light vanishes through the medium in the absence of a magnetic field (the MO rotation of the polarization plane changes with changing the magnetic field, and we notice $25^\circ$ at $\Delta B=\pm 2.5 \gamma$ for $\phi =\frac{\pi }{2}$ . The MO rotation of the polarization plane reverses completely by $180^\circ$ by considering the relative phase $\phi =\frac{3 \pi }{2}$ in the system. The enhanced and almost double MO rotation is observed through Doppler broadened medium for the same set of parameters, as in Fig. 2.

**Figure 5:**
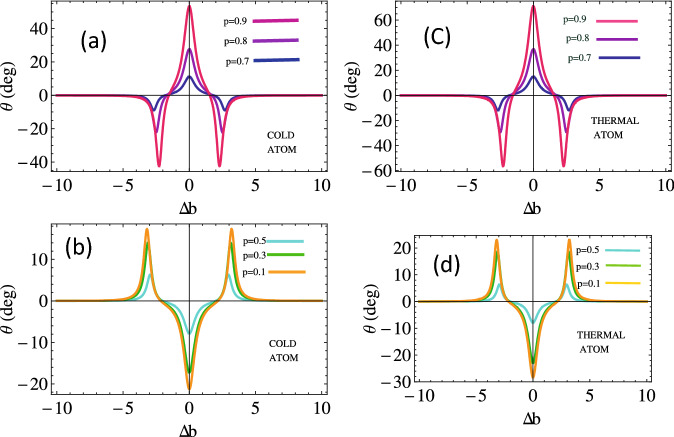
Magneto-optical rotation versus probe field detuning for cold atom *kD* = 0 (**a**), (**c**) and thermal atom $kD = 50 \gamma$ (**b**), (**d**) w.r.t SGC parameter. The other parameters are $\Omega _{1}=2.5,\Omega _{k}=2, p=0.9, \Delta _{p}=0$.

Moreover, we investigate magneto-optical (MO) rotation of the left and right handed light in cold and Doppler broadened double lambda atomic medium. We measure the rotation angle as a function of the probe detuning for various strengths of SGC parameter *p* as depicted in Fig. 4. We notice that the MO polarization plane rotates anti-clockwise with the increasing $\Delta {p}$ up to 5. The enhanced effect is observed for Doppler’s broadened medium (see Fig. 4b). There is no polarization plane rotation of the probe light at $p=0.6$. The polarization plane switches to clockwise rotation as the SGC parameter increases beyond 0.6 and we observe a $50^\circ$ rotation of the polarization plane for $p=0.9$ in the cold medium. We notice almost 1.4 times enhancement in the MO rotation of the probe in Doppler broadened medium (see Fig. 4d).

**Figure 6:**
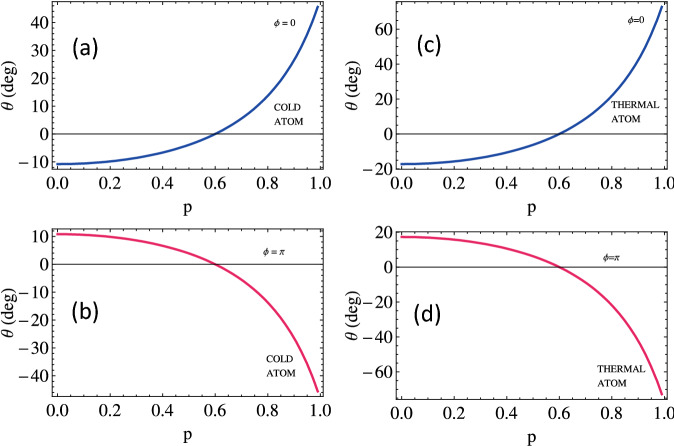
Magneto-optical rotation versus SGC parameter for cold atom *kD* = 0 (**a**), (**c**) and thermal atom $kD = 50 \gamma$ (**b**), (**d**) w.r.t relative phase, $\Omega _{1}=2.5$, $\Omega _{k}=2, \phi =0, \Delta _{p}=0$. The other parameters are same as in Fig. 2.

Now, to present the behavior of rotation angle with magnetic field for various choices of interference parameter *p* with and without Doppler broadening medium. We depict rotation angle with $\Delta b$, as shown in Fig. 5. We notice that the MO rotation of the handed light in the Doppler-free medium increases in the clockwise direction with the increasing value of *p* beyond 0.6, as plotted in Fig. 5a. The enhanced MO rotation of the polarization plane up to 65 degrees through the Doppler broadening atomic medium is observed, which is 1.3 times greater than that in the case of a cold atom (see Fig. 5b). The rotation angle of the polarization plane reverses its direction through the Doppler-free medium by keeping the inference term relatively small. We observe a $20^\circ$ clockwise rotation angle through the Doppler-free medium and 30 degrees through the Doppler broadened medium in the resonance condition for $p=0.1$. Thus Doppler broadened medium can be used to enhance the clock or anti-clockwise MO rotation and has potential application in magnetometery^54^.

**Figure 7:**
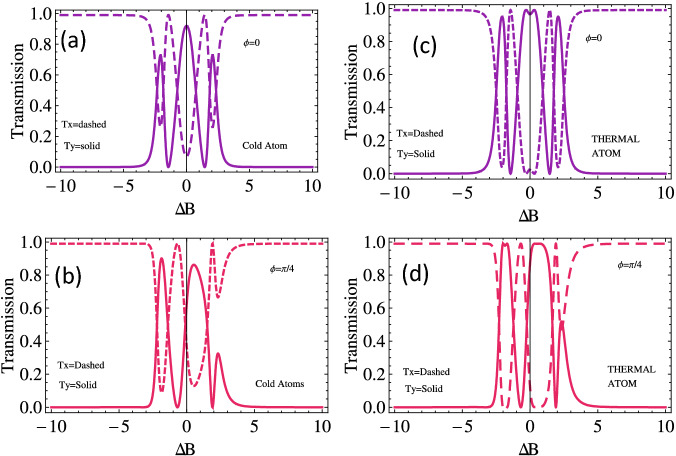
Transmission intensity along *x* and *y*-axis versus SGC parameter for cold atom *KD* = 0 (**a**), (**c**) and thermal atom $KD = 50 \gamma$ (**b**), (**d**) at $\phi =0$ and $\phi =\pi$. The other parameters are $\Omega _{1}=2.5, \Omega _{k}=2$, $\Delta _{p}=0$. The other parameters are same as in Fig. 2.

We present the study of the MO rotation with SGC parameter through Doppler’s free and Doppler’s broadened medium for different values of relative phases. Initially, when there is no phase difference, the MO rotation of the polarization plane increases from $-10^\circ$ to $+50^\circ$ through Doppler-free medium and $-20^\circ$ to $+70^\circ$ through Doppler broadened medium (see Fig. 6a,b) with increasing SGC. The MO rotation of the polarization plane completely reverses through Doppler-free and Doppler broadened medium when the phase angle changes from 0 to $\pi$, as plotted in Fig. 6c,d. For this case, we observe a clockwise rotation increasing from $+10^\circ$ to $-40^\circ$ for cold media while enhanced rotation from $+20^\circ$ to $-80^\circ$ is observed in case of thermal media. The polarization plane of the probe light remains unchanged through the medium at $\mathrm {p}=0.6$. Thus, the quantum interference term can be used as the knob to change the polarization from clock to anticlockwise direction.

**Figure 8:**
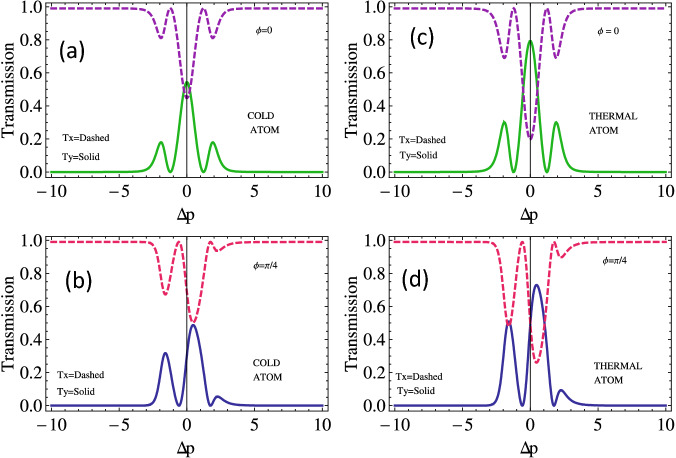
The output field’s intensity (**a**) in the *y*-direction $T_{y}$ and in the *x*-direction $T_{x}$ versus magnetic field detuning for varying relative phase. The other parameters assumed are the same as used in Fig. 2.

To investigate the behavior of the output or transmission of left and right handed light through Doppler-free and broadened medium with magnetic field detuning for different choices relative phase. We plot $T_{x}$ and $T_{y}$ versus $\Delta b$, plotted in Fig. 7. Initially, when all the applied fields possess the same phase $(\phi =0)$, the transmission of the light through Doppler-free medium along the *x*-axis is almost zero at zero magnetic field detuning and observe an enhanced transmission at $\Delta b=\pm 1.5 \gamma$ (see dotted line in Fig. 7a). The probe field is transmitted through the Doppler-free medium along *y* axis at resonance condition and gets absorbed at $\Delta b=\pm 1.5 \gamma$. Similar enhanced behavior is observed in the output probe light in the Doppler’s broadened medium along the *x*-axis and *y*-axis (see Fig. 7b). The output probe light along the axis through the Doppler-free medium transmit at $\Delta b=-1 \gamma$ and $2 \gamma$ when the relative phase is $\frac{\pi }{4}$ (see the dashed pink curve of Fig. 7c), while the light pulse almost passes along the $\mathrm {y}$ axis $\Delta b=1 \gamma$ and $-2 \gamma$. Enhanced similar behavior of the out pulse is observed for both $T_x$ and $T_y$ through the Doppler broadened medium for the same relative phase (see Fig. 7d).

To investigate the transmission of probe light through the cold and thermal double lambda atomic medium along *x* and *y* axes, we plot the transmission of the left and right handed light with probe detuning $(\Delta _p)$ for various values of relative phases, as shown in Fig. 8. The transmission along both the *x*-axis and the *y*-axis is reported to be 0.55 and 0.45, respectively, in the Doppler-free medium at the resonance condition when the relative phase is zero, see Fig. 8a. The transmission spectrum $T_y$ increases further to 0.8, and $T_x$ decreases to 0.2 in the Doppler broadened medium at the resonance condition, as presented in Fig. 8c. The transmission peak of the light along the *y*-axis and absorption in the *x*-direction through the Doppler medium shift towards positive probe detuning when the relative phase difference is $45^\circ$, see Fig. 8b. An enhanced behavior of the transmission spectrum of the handed light along both axes is observed through Doppler broadened medium, see Fig. 8d.

**Figure 9:**
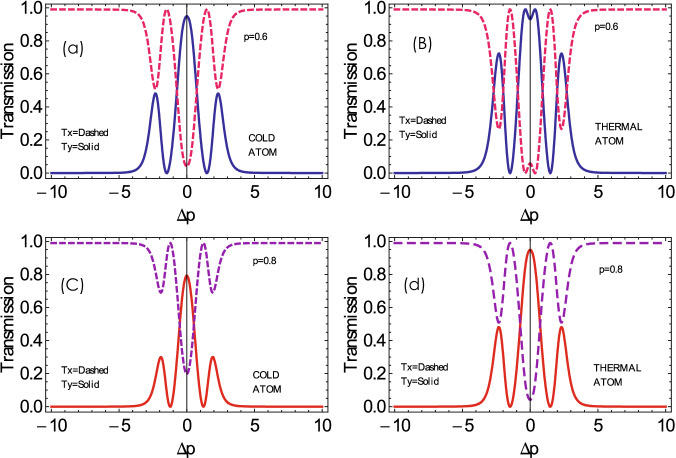
The output field intensity of the (**a**) along *y*-axis ($T_y$) and along *x*-axis ($T_x$) versus probe field detuning for varying values of SGC parameter. The other parameters selected are the same as that of Fig. 2.

**Figure 10:**
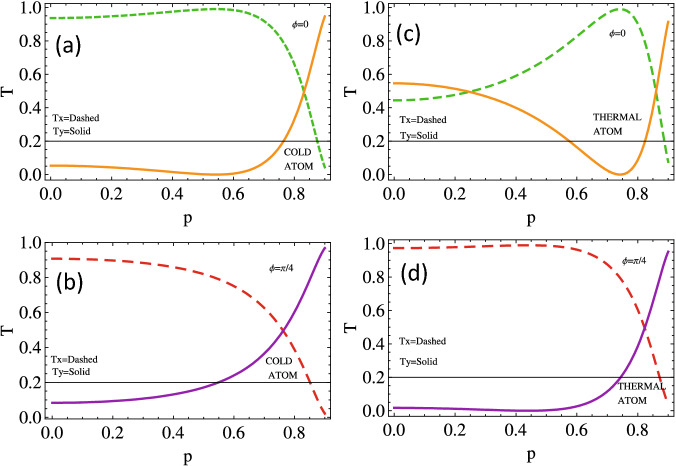
The intensity of the output field in *y* and *x* directions against the SGC parameter. The other parameters are the same used in Fig. 2.

**Figure 11:**
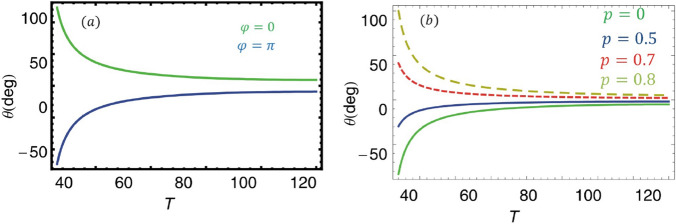
Magneto-optical rotation versus probe temperature of the sample, T such that $\Omega _{1}=2.5, \Omega _{k}=2$, $\Delta _{p}=0, \Delta _{b}=10 \gamma$ and $p=0.8$ in (**a**), and $\phi$ =0 in (**b**). The other parameters are kept the same as in Fig. 2.

We study the transmission intensity of the left and right handed light with probe detuning for the different values of interference, i.e., SGC *p* in the system. We notice that transmission of the light along the *x*-axis is almost 0.1 and 0.9 along the *y*-axis in cold atoms, at resonance point for $\mathrm {p}=0.6$ (see Fig. 9a). Multiple enhanced out field peaks of the left and right handed lights along both the axes are noticed in the Doppler broadened medium by considering moderate SG coherence in the system (see Fig. 9c). The transmission peaks of the left and right handed polarized probe light reduce to a single transmission peak by increasing the SGC effect in the medium (see Fig. 9b, d). The transmission of the left and right handed polarized probe light increases in the Doppler broadened medium by taking a high SGC effect in the system, as shown in Fig. 9d. We observe transmission along y-axis to be enhanced from 0.8 to 0.9 in thermal atomic media and transmission along *x*-axis to be reduced up-to nearly zero simultaneously, as shown in Fig. 9d.

To present the transmission of the probe field through the Doppler-free and Doppler-broadened medium with SGC parameter for different values of relative phases, we plot $T_x$ and $T_y$ versus *p*, as depicted in Fig. 10. The transmission of the probe light along the *x* direction $T_x$ is maximum while minimum along the *y* direction $T_y$ with increasing *p* up to 0.6 through Doppler-free medium and then start decreasing along the *x*-axis and increasing sharply along the *y* axis with the increasing *p* from 0.6 to 0.99 The probe light partially absorbs and transmits for almost all values of $\mathrm {p}$ in both $\mathrm {x}$ and $\mathrm {y}$ direction but transmitted completely along the *x*-axis and absorbed along the *y*-axis at $p=0.75$ through the Doppler broadened medium (see sub-figure d). The polarized light through the Doppler-free medium for relative phase $\frac{\pi }{4}$ partially transmitted in both directions but completely absorbed along *x*-axis while transmitted completely at $p=0.99$, as in Fig. 10c. The transmission and absorption of the probe light are constant along *x* and *y* axes up to 0.6 respectively, and then dramatically changes in the reverse direction from $p=0.6$ and onward.

To present a detailed study of the MOR of the polarized light versus temperature T, we show a plot between rotation angle and T with varying relative phases and SE coherence. Initially, when all the medium fields are in phase, the rotational angle is 100 degrees at room temperature and then decreases gradually with temperature, as illustrated in Fig. 11a. A similar but opposite rotational angle is observed as the relative phase reaches $\pi$. Figure 11b describes the MOR of the polarized light against temperature by varying SEC in the medium. The polarized light rotates anti-clockwise when no SE coherence is considered in the medium. The gradual shift of the MOR to clockwise is noticed by increasing the SEC parameter in the system. We observe the enhancement in rotational angle (positive and negative) at the room temperature and it degrades as the temperature of the system increases and eventually reaches zero. The anisotropic nature of the medium is lost at high temperatures because both the left/right dispersion susceptibilities become equal, and was previously observed in Ref.^55^.

## Conclusions

In this article, we studied MOR and transmission of the probe field through an optically active thermal and cold medium containing double-lambda closed-loop atomic medium with varying magnetic field. The relative phase of the coupling fields not only played a significant role in enhancing and diminishing the rotation of the circularly polarized beams, but also in tuning the MOR form clock to an anti-clockwise direction through the Doppler broadened medium. We noticed $+100^\circ$ ($-100^\circ$) of the rotation angle when all the fields are in phase (out of phase) in medium with thermal atoms. Similarly, the MOR of the left/right-handed polarized light can be tuned (clock and anticlockwise) and enhanced by changing SG coherence in the system. The polarization plane rotation of the polarized lights remained unaffected through the system when SGC parameter is specifically 0.6. Multiple enhanced outfield peaks of the left and right handed lights along both the axes are noticed in the Doppler broadened medium by considering moderate SG coherence in the system. The transmission peaks of the left and right handed probe lights are reduced to a single transmission peak when increased SGC effect in the thermal medium. We further found that the anisotropy of the medium is lost at higher temperatures. Our scheme can be effectively used at room temperature. Polarization spectroscopy, precision measurements, polarization converter of the TE/TM modes in optical communications, and depolarization backscattering lidar are some applications of our findings.

## Data Availability

All data generated or analyzed during this study are included in this manuscript.
